# Redetermination of tantalum penta­bromide, (TaBr_5_)_2_

**DOI:** 10.1107/S1600536810032538

**Published:** 2010-08-21

**Authors:** Katja Habermehl, Ingo Pantenburg, Gerd Meyer

**Affiliations:** a Universität zu Köln, Institut für Anorganische Chemie, Greinstrasse 6, D 50939 Köln, Germany

## Abstract

Crystals of di-μ-bromido-bis­[tetra­bromidotantalum(V)], (TaBr_5_)_2_, were obtained by recrystallization at 773 K. A first crystal structure study of (TaBr_5_)_2_ was reported by Rolsten [*J. Am. Chem. Soc.* (1958) ▶, **80**, 2952–2953], who analysed the powder diffraction pattern and came to the conclusion that it crystallizes isotypically with (NbBr_5_)_2_ in a primitive ortho­rhom­bic cell. These findings are not in agreement with our current results of a monoclinic *C*-centred structure. (TaBr_5_)_2_ is isotypic with α-(NbCl_5_)_2_. The crystal structure contains [TaBr_6_] octa­hedra sharing common edges forming [TaBr_5_]_2_ dimers. Two crystallographically independent dimers with symmetries *m* and 2/*m* and Ta⋯Ta distances of 4.1574 (11) and 4.1551 (15) Å, respectively, are present in the structure.

## Related literature

For a previous study of (TaBr_5_)_2_, see: Rolsten (1958*a* ▶), who also reported the crystal structure of (NbBr_5_)_2_ (Rolsten, 1958*b* ▶). (TaBr_5_)_2_ is isotypic with α-(NbCl_5_)_2_, the structure of which was first described by Zalkin & Sands (1958 ▶) and was redetermined by Hoenle & von Schnering (1990 ▶). For a summary of all possible stackings of double octa­hedral mol­ecules in penta­halides of Nb and Ta, see: Müller (1978 ▶). Experimental details can be found in Brauer’s handbook (Brauer, 1962 ▶). For data analysis, see: Spek (2009 ▶).

## Experimental

### 

#### Crystal data

Ta_2_Br_10_
*M* *_r_* = 1161.00
Monoclinic, 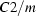

*a* = 19.433 (3) Å
*b* = 18.775 (2) Å
*c* = 6.2034 (10) Å
β = 90.716 (13)°
*V* = 2263.2 (6) Å^3^
*Z* = 6
Mo *K*α radiation
μ = 40.93 mm^−1^
*T* = 293 K
0.14 × 0.09 × 0.04 mm

#### Data collection

Stoe IPDS 2 diffractometer
Absorption correction: numerical (*X-SHAPE*; Stoe & Cie, 1999 ▶) *T*_min_ = 0.014, *T*_max_ = 0.089
17962 measured reflections
2605 independent reflections
1678 reflections with *I* > 2σ(*I*)
*R*_int_ = 0.125

#### Refinement

*R*[*F* ^2^ > 2σ(*F* ^2^)] = 0.042
*wR*(*F* ^2^) = 0.109
*S* = 0.95
2605 reflections
88 parameters
Δρ_max_ = 1.71 e Å^−3^
Δρ_min_ = −1.53 e Å^−3^

### 

Data collection: *X-AREA* (Stoe & Cie, 2002 ▶); cell refinement: *X-RED* (Stoe & Cie, 2002 ▶); data reduction: *X-RED*; program(s) used to solve structure: *SIR92* (Altomare *et al.*, 1994 ▶); program(s) used to refine structure: *SHELXL97* (Sheldrick, 2008 ▶); molecular graphics: *DIAMOND* (Brandenburg, 2005 ▶); software used to prepare material for publication: *SHELXL97*.

## Supplementary Material

Crystal structure: contains datablocks I, global. DOI: 10.1107/S1600536810032538/wm2384sup1.cif

Structure factors: contains datablocks I. DOI: 10.1107/S1600536810032538/wm2384Isup2.hkl

Additional supplementary materials:  crystallographic information; 3D view; checkCIF report

## Figures and Tables

**Fig. 1.**
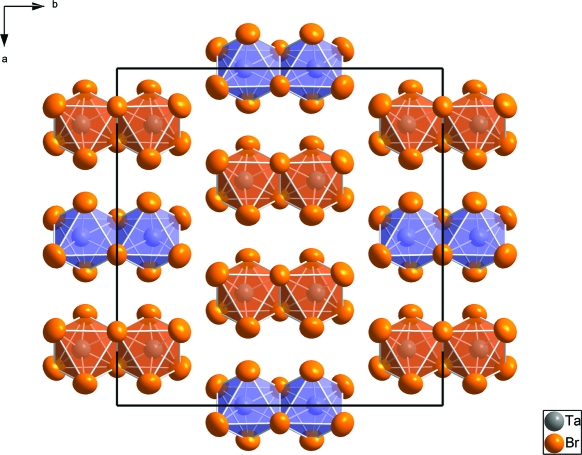
Unit cell of TaBr5 in a view along [001]. Double octahedral molecules are coloured in orange or blue for depth perception. Thermal ellipsoids are given on the 99% probability level.

## supplementary crystallographic information

### Comment

(TaBr_5_)_2_ was first described by Rolsten (Rolsten, 1958*a*). He compared the powder diffraction pattern of (TaBr_5_)_2_ with orthorhombic NbBr_5_ (space group *Pbam*) and came to the conclusion that the structures are alike. The structure of (NbBr_5_)_2_ was already known from single-crystal structure analysis (Rolsten, 1958*b*). However, the apparent isotypism between (TaBr_5_)_2_ and (NbBr_5_)_2_ is not in agreement with our result that shows (TaBr_5_)_2_ to crystallize in the monoclinic space goup *C*2/*m*.

The crystal structures of all pentahalides besides the fluorides contain [*MX*_6_] octahedra (*M* = Nb, Ta; *X* = Cl, B, I) sharing common edges forming [*MX*_5_]_2_ dimers. These double octahedra can be stacked in different ways, resulting in different structure types. In the title compound, the stacking of the (TaBr_5_)_2_ layers along *b* can be described as *A*_1_*B*_1_*A*_1_*B*_1_··· . Within one layer the molecules are stacked in a "2 1" stacking scheme (Fig. 1). A summary of all possible stacking possibilities has been given by Müller (1978). The [TaBr_6_] octahedra are distorted due to the repulsive forces of the highly charged metal atoms centering the octahedra with *d*(Ta—Ta) = 4.1574 (11) Å for the (Ta1Br_5_)_2_ dimer (*m* symmetry) and 4.1551 (15) Å for the (Ta2Br_5_)_2_ dimer (2/*m* symmetry).

(TaBr_5_)_2_ is isotypic with α-(NbCl_5_)_2_ (Zalkin & Sands, 1958; Hoenle & von Schnering, 1990).

### Experimental

TaBr_5_ was synthesized according to an experimental procedure reported in Brauer's handbook (Brauer, 1962). Orange polyhedric crystals were obtained by recrystallization of TaBr_5_ in a silica ampoule. The ampoule was heated with 50 K per hour to 773 K and annealed for 12 h, after which it was slowly cooled to room temperature with 2 K per hour. Single crystals of TaBr_5_ were selected under a microscope in an argon-filled glove box.

### Refinement

*PLATON* (Spek, 2009) indicates higher (pseudo)-symmetry and suggests a change of the crystal system from monoclinic *C* to orthorhombic *C*, but the experimentally determined unit cell angles differ with 0.72° considerably from orthogonality. The maximum residual density lies 1.29Å from Ta1 and the density minimum lies at the Br4 atom.

### Crystal data

**Table:**

Ta_2_Br_10_	*F*(000) = 2976
*M**_r_* = 1161.00	none
Monoclinic, *C*2/*m*	*D*_x_ = 5.111 Mg m^−^^3^
Hall symbol: -C 2y	Mo *K*α radiation, λ = 0.71073 Å
*a* = 19.433 (3) Å	Cell parameters from 7929 reflections
*b* = 18.775 (2) Å	θ = 2.1–27.1°
*c* = 6.2034 (10) Å	µ = 40.93 mm^−^^1^
β = 90.716 (13)°	*T* = 293 K
*V* = 2263.2 (6) Å^3^	Polyhedron, orange
*Z* = 6	0.14 × 0.09 × 0.04 mm

### Data collection

**Table:**

Stoe IPDS 2 diffractometer	2605 independent reflections
Radiation source: fine-focus sealed tube	1678 reflections with *I* > 2σ(*I*)
graphite	*R*_int_ = 0.125
Detector resolution: 0 pixels mm^-1^	θ_max_ = 27.3°, θ_min_ = 1.5°
oscillation scans	*h* = −24→24
Absorption correction: numerical (*X-SHAPE*; Stoe & Cie, 1999)	*k* = −24→24
*T*_min_ = 0.014, *T*_max_ = 0.089	*l* = −7→7
17962 measured reflections	

### Refinement

**Table:**

Refinement on F^2^	Primary atom site location: structure-invariant direct methods
Least-squares matrix: full	Secondary atom site location: difference Fourier map
R[F^2^ > 2σ(F^2^)] = 0.042	w = 1/[σ^2^(F_o_^2^) + (0.0563P)^2^] where P = (F_o_^2^ + 2F_c_^2^)/3
wR(F^2^) = 0.109	(Δ/σ)_max_ = 0.001
S = 0.95	Δρ_max_ = 1.71 e Å^−^^3^
2605 reflections	Δρ_min_ = −1.53 e Å^−^^3^
88 parameters	Extinction correction: SHELXL97 (Sheldrick, 2008), Fc^*^=kFc[1+0.001xFc^2^λ^3^/sin(2θ)]^-1/4^
0 restraints	Extinction coefficient: 0.00064 (4)
0 constraints	

### Special details

**Table:**

Geometry. All e.s.d.'s (except the e.s.d. in the dihedral angle between two l.s. planes) are estimated using the full covariance matrix. The cell e.s.d.'s are taken into account individually in the estimation of e.s.d.'s in distances, angles and torsion angles; correlations between e.s.d.'s in cell parameters are only used when they are defined by crystal symmetry. An approximate (isotropic) treatment of cell e.s.d.'s is used for estimating e.s.d.'s involving l.s. planes.
Refinement. Refinement of *F*^2^ against ALL reflections. The weighted *R*-factor *wR* and goodness of fit *S* are based on *F*^2^, conventional *R*-factors *R* are based on *F*, with *F* set to zero for negative *F*^2^. The threshold expression of *F*^2^ > σ(*F*^2^) is used only for calculating *R*-factors(gt) *etc*. and is not relevant to the choice of reflections for refinement. *R*-factors based on *F*^2^ are statistically about twice as large as those based on *F*, and *R*- factors based on ALL data will be even larger.

### Fractional atomic coordinates and isotropic or equivalent isotropic displacement parameters (Å^2^)

**Table:**

	*x*	*y*	*z*	*U*_iso_*/*U*_eq_	
Ta1	1.0000	0.38934 (4)	0.5000	0.0296 (2)	
Ta2	0.66677 (3)	0.38928 (3)	0.97909 (7)	0.02938 (18)	
Br1	0.61422 (10)	0.5000	1.2034 (3)	0.0326 (4)	
Br2	0.61078 (9)	0.30802 (8)	1.2210 (2)	0.0430 (4)	
Br3	0.56469 (8)	0.40167 (8)	0.7436 (2)	0.0406 (3)	
Br4	0.72279 (9)	0.30797 (8)	0.7378 (2)	0.0430 (4)	
Br5	0.71903 (11)	0.5000	0.7544 (3)	0.0321 (4)	
Br6	0.89789 (8)	0.40192 (8)	0.7271 (2)	0.0421 (3)	
Br7	0.76900 (8)	0.40188 (8)	1.2143 (2)	0.0415 (3)	
Br8	1.05614 (9)	0.30768 (8)	0.7446 (2)	0.0451 (4)	
Br9	1.05245 (11)	0.5000	0.7294 (3)	0.0323 (4)	

### Atomic displacement parameters (Å^2^)

**Table:**

	*U*^11^	*U*^22^	*U*^33^	*U*^12^	*U*^13^	*U*^23^
Ta1	0.0289 (4)	0.0278 (4)	0.0320 (4)	0.000	0.0032 (3)	0.000
Ta2	0.0292 (3)	0.0280 (3)	0.0310 (3)	0.0002 (2)	0.00055 (19)	−0.00014 (18)
Br1	0.0353 (11)	0.0314 (9)	0.0312 (8)	0.000	0.0070 (7)	0.000
Br2	0.0464 (10)	0.0408 (8)	0.0419 (7)	−0.0076 (6)	0.0083 (6)	0.0062 (5)
Br3	0.0338 (8)	0.0460 (8)	0.0419 (7)	−0.0012 (6)	−0.0074 (5)	−0.0021 (5)
Br4	0.0465 (10)	0.0396 (7)	0.0431 (7)	0.0060 (7)	0.0079 (6)	−0.0087 (6)
Br5	0.0343 (11)	0.0316 (9)	0.0305 (8)	0.000	0.0067 (7)	0.000
Br6	0.0353 (8)	0.0457 (8)	0.0455 (7)	−0.0017 (6)	0.0125 (6)	0.0023 (6)
Br7	0.0349 (8)	0.0453 (8)	0.0440 (7)	0.0008 (6)	−0.0079 (5)	0.0031 (6)
Br8	0.0464 (10)	0.0400 (7)	0.0487 (8)	0.0075 (7)	−0.0038 (6)	0.0096 (6)
Br9	0.0360 (11)	0.0305 (8)	0.0303 (8)	0.000	−0.0039 (7)	0.000

### Geometric parameters (Å, °)

**Table:**

Ta1—Br8^i^	2.4087 (15)	Ta2—Br3	2.4598 (16)
Ta1—Br8	2.4087 (15)	Ta2—Br7	2.4613 (17)
Ta1—Br6	2.4591 (14)	Ta2—Br5	2.7075 (12)
Ta1—Br6^i^	2.4591 (14)	Ta2—Br1	2.7084 (12)
Ta1—Br9	2.7100 (13)	Br1—Ta2^iii^	2.7084 (12)
Ta1—Br9^ii^	2.7100 (13)	Br5—Ta2^iii^	2.7075 (12)
Ta2—Br4	2.4075 (14)	Br9—Ta1^ii^	2.7100 (13)
Ta2—Br2	2.4092 (15)		
			
Br8^i^—Ta1—Br8	100.93 (9)	Br2—Ta2—Br3	93.59 (6)
Br8^i^—Ta1—Br6	93.41 (6)	Br4—Ta2—Br7	93.53 (6)
Br8—Ta1—Br6	93.60 (6)	Br2—Ta2—Br7	93.39 (6)
Br8^i^—Ta1—Br6^i^	93.60 (6)	Br3—Ta2—Br7	169.06 (5)
Br8—Ta1—Br6^i^	93.41 (6)	Br4—Ta2—Br5	89.51 (5)
Br6—Ta1—Br6^i^	168.98 (8)	Br2—Ta2—Br5	169.14 (5)
Br8^i^—Ta1—Br9	169.48 (6)	Br3—Ta2—Br5	85.79 (6)
Br8—Ta1—Br9	89.59 (5)	Br7—Ta2—Br5	85.77 (6)
Br6—Ta1—Br9	85.78 (6)	Br4—Ta2—Br1	169.22 (5)
Br6^i^—Ta1—Br9	85.77 (6)	Br2—Ta2—Br1	89.43 (5)
Br8^i^—Ta1—Br9^ii^	89.59 (5)	Br3—Ta2—Br1	85.76 (6)
Br8—Ta1—Br9^ii^	169.48 (6)	Br7—Ta2—Br1	85.90 (6)
Br6—Ta1—Br9^ii^	85.77 (6)	Br5—Ta2—Br1	79.71 (4)
Br6^i^—Ta1—Br9^ii^	85.78 (6)	Ta2—Br1—Ta2^iii^	100.26 (6)
Br9—Ta1—Br9^ii^	79.90 (6)	Ta2—Br5—Ta2^iii^	100.31 (6)
Br4—Ta2—Br2	101.35 (6)	Ta1—Br9—Ta1^ii^	100.10 (6)
Br4—Ta2—Br3	93.34 (6)		

Symmetry codes: (i) −*x*+2, *y*, −*z*+1; (ii) −*x*+2, −*y*+1, −*z*+1; (iii) *x*, −*y*+1, *z*.
